# Effectiveness of Nitrous Oxide versus Pethidine/Midazolam for Pain Relief in Minor Gynecological Operative Procedures: A Randomized Controlled Trial

**DOI:** 10.3390/medicina59030611

**Published:** 2023-03-20

**Authors:** Napas Lohtrakul, Chanane Wanapirak, Theera Tongsong

**Affiliations:** Department of Obstetrics and Gynecology, Faculty of Medicine, Chiang Mai University, Chiang Mai 50200, Thailand

**Keywords:** curettage, Entonox^®^, gynecological procedure, nitrous oxide, midazolam, pethidine

## Abstract

*Aim and Objective:* To compare the analgesic effectiveness of the patient-controlled inhaled nitrous oxide (Entonox^®^) with intravenous opioids (pethidine/midazolam) in reducing pain during minor gynecological operative procedures, including manual vacuum aspiration (MVA), fractional curettage and dilatation and curettage. *Materials and Methods:* Patients undergoing minor gynecological procedures from August 2021 to December 2022 were randomized to receive nitrous oxide or intravenous pethidine (50–75 micrograms) plus midazolam (2 mg). Pain scores during and post-procedure, satisfaction level, and side effects were assessed and compared. *Results:* A total of 106 patients met the inclusion criteria, including 53 in the pethidine/midazolam group and 53 in the nitrous oxide group. Baseline characteristics were comparable (*p*-value > 0.05). Pain scores during, immediately and 30 min after procedures were not significantly different in two groups (4.94 ± 3.15, 2.74 ± 2.57, 1.58 ± 2.13 vs. 5.47 ± 2.80, 2.98 ± 2.70, 1.64 ± 2.70; *p*-value: 0.174, 0.634, 0.889, for pethidine/midazolam vs. nitrous oxide group, respectively. Satisfaction scores were comparable in both groups (*p*-value > 0.05). However, the rate of side effects was significantly lower in the nitrous oxide group (3.8% vs. 28.3%; *p*-value 0.001). Additionally, the discharge scores showed a significantly faster recovery time in the nitrous oxide group at 60 and 90 min after the procedure; median (IQR): 10 (9–10) vs. 9 (8–10) and 10 (10–10) vs. 10 (8.5–10); *p*-value 0.002 and 0.029, respectively). *Conclusions:* Nitrous oxide is as effective as pethidine/midazolam for pain relief in minor gynecological operative procedures but associated with significantly lower side effects and significantly faster recovery time.

## 1. Introduction

Minor gynecological operative procedures, including dilatation curettage, fractional curettage, or manual vacuum aspiration, are commonly performed in daily gynecologic practice. Typically, the device is inserted through the cervical canal into the uterine cavity to obtain the tissue for diagnosis or treatment. Common indications for such procedures include abnormal uterine bleeding, termination of early pregnancy, evacuation of miscarriages, and retained pieces of the placenta or conceptive products. Although these are brief procedures lasting for 10–15 min and can be performed in an outpatient setting, the procedures are often associated with significant pain or uterine cramps during curettage, similar to labor pain. Therefore, pain management is an important component of the procedures for successful outcomes without complications.

Intravenous opioid derivatives and benzodiazepines are commonly used for pain management during a minor gynecological operative procedure. In addition, in our practice, we usually use pethidine (meperidine) for pain control as a method of choice because it is inexpensive, available, and has a rapid onset of action in about 5 min. Typically, pethidine combined with midazolam or diazepam, a benzodiazepine for anxiolysis, releases an effect in about 45 min with a duration of action of up to 2–4 h. Nevertheless, pethidine/midazolam is often associated with several side effects, such as respiratory depression, tachycardia, hypotension, and gastrointestinal symptoms. Additionally, Entonox^®^ is more convenient to administer than intramuscular injection of pethidine.

Currently, Entonox^®^, a mixture of 50% nitrous oxide (N_2_O) and 50% oxygen, used as a self-controlled inhaled gas for pain control, has become widely available. Entonox^®^ provides analgesic, anxiolytic, and amnesic effcts [1]. Since it has a rapid onset of action and quick recovery time, it is beneficial and suitable for patients undergoing short surgical procedures. In addition, in clinical practice, nitrous oxide sedation has widely been used in other specialties, including dentistry [2], emergency medicine [3], and colonoscopy [4], and is commonly used to control labor pain [5]. For example, Mobarak et al. [6] demonstrated that, in a randomized controlled trial, Entonox^®^ provided better pain relief for labor pain compared to a single dose of pethidine 30 min after intervention. Nevertheless, studies on nitrous oxide for pain relief in gynecologic procedures have been published in a very limited number. Del Valle Rubido et al. [7] demonstrated in their pilot study that nitrous oxide was a safe and effective analgesic technique for polypectomy office hysteroscopy compared with the paracervical infiltration and control groups. Accordingly, we conducted this randomized controlled trial aimed to compare the analgesic effectiveness of the patient-controlled inhaled nitrous oxide (Entonox^®^) with intravenous opioids (pethidine and midazolam) in reducing pain during minor gynecological operative procedures, including fractional curettage, dilatation and curettage and manual vacuum aspiration (MVA).

## 2. Patients and Methods

This study is a single-center, prospective, randomized, open-label controlled trial in two parallel arms to compare the analgesic efficacy of the patient-controlled inhaled nitrous oxide with pethidine/midazolam at Maharaj Nakorn Chiang Mai hospital, Chiang Mai University, Thailand (a tertiary center, medical teaching school) between August 2021 to December 2022. The study was ethically approved by the Institutional Review Board (Research ID: OBG-2564-08098). The project was registered for clinical control trial study at the Thai Clinical Trials Registry (TCTR20230107003). All of the participants were invited to participate in the study and were systematically informed about the objective and intervention of the research. Written informed consent was provided by all participants. The study population was gynecologic patients undergoing minor gynecologic procedures in an outpatient setting. The women met the following inclusion criteria: (1) age of greater than 18 years; (2) indicated for minor surgical procedures including dilatation and curettage, fractional curettage, and manual vacuum aspiration (MVA); (3) understanding of the study and capable of self-controlled inhaled nitrous oxide administration. The exclusion criteria were as follows: (1) unstable hemodynamics; (2) contraindicated for medications used in the study, such as the history of severe respiratory or cardiac disease, thyroid disease, neurological disorder, renal disease, upper respiratory infections or sinus blockage, the recent history of middle or inner ear surgery, pneumothorax or intestinal obstruction, and ASA physical status III/IV; (3) patients with a history of a previous gynecologic procedure such as curettage.

All participants were randomly allocated to one of the two groups: the group using nitrous oxide and the group using pethidine/midazolam for pain relief during the procedure. A randomization scheme was prepared and organized by one of the authors (NL) prior to the beginning of the study, and the code for each participant was kept in a sealed, black opaque envelope. The randomization was a ratio of one-to-one, using a computer-generated block randomization sequence with a random sequence with a block size of 4. This was an open-label controlled trial. The patients, care providers, and study investigators were not blinded to the study group. After randomization, the women could withdraw from the study at any time.

***Study protocol:*** Baseline characteristics, including obstetric and gynecologic history as well as indication for the procedures, were prospectively collected and recorded in a research form. After enrollment and randomized allocation, the participants in the pethidine/midazolam group were given 50 mg of pethidine and 2 mg of midazolam intravenously 5 min before starting the procedure, whereas those in the nitrous oxide group were instructed to begin inhalation via mouthpiece 2 min before starting the procedure and continued inhaling gas when she felt pain or until the end of the procedure. The participants in the nitrous oxide group were trained for the self-administration of Entonox^®^ by the nurse who was in charge of that case. If the participants needed more pain control during the procedure, those in the intravenous sedation group received an additional 25–50 mg of pethidine, and those in the nitrous oxide group received 50 mg of intravenous pethidine and 2 mg of midazolam after discontinuation of the nitrous oxide. The participants who received both intravenous sedation and nitrous oxide were not excluded from the study and were included in the analysis as intention-to-treat.

The minor gynecological operative procedures were performed by well-trained gynecologists. The procedures followed the clinical standard of care and were not altered for this study. All participants were assessed for pain scores during, immediately post-procedure, and 30 min post-procedure, satisfactory levels, and side effects. Immediately after the procedure and while still receiving the allocated medication, participants rated their maximum pain on the Numerical Rating Scale (NRS). The NRS is a 10-point scale with a score of 0 reflecting “no pain” and a score of 10 reflecting “maximum pain”. At 30 min post-procedure, participants completed another NRS, recalling maximum pain during the procedure and satisfactory level. Participants were monitored at 60, 90, and 120 min post-procedure or until they achieved a modified post-anesthesia discharge scoring system of 9 or greater, indicating recovery from sedation. However, according to our hospital protocol, all patients were discharged from the hospital at 2 h after the procedure.

***Outcome measures:*** The primary outcome measure was the difference between the two groups in maximum pain during, immediately post-procedure, and 30 min post-procedure as measured on NRS. The secondary outcomes were satisfactory level, side effects, and discharge time. The patient’s pain level during the procedure and post-procedure, along with the satisfaction levels and the presence or absence of side effects, were assessed after the procedure. Regarding the discharge time evaluation, the modified post-anesthesia discharge scoring system was used to assess at 60, 90, and 120 min post-procedure. A score of 9 or greater was considered as complete recovery and ready to discharge home.

***Statistical analysis:*** The data analysis was performed using the statistical package for the social sciences (SPSS) software version 26.0 (IBM Corp. Released 2019. IBM SPSS Statistics for Windows, Version 26.0 IBM Corp: Armonk, NY, USA). The baseline demographic data were presented as mean + SD or median (IQR) for continuous variables as appropriate and as a percentage for the categorical variables. In comparisons of the data between both groups, Student’s *t*-tests, Mann–Whitney U, and Cchi-square were applied as appropriate. A *p*-value of less than 0.05 was regarded as statistical significance. According to the primary outcome, this study as a non-inferiority trial needed a sample size of at least 84 cases, 42 in each group, to gain a power of 90% at a 95% confidence interval when a reduction in visual analog pain score of 1 was considered clinically significant.

## 3. Results

**Figure 1:**
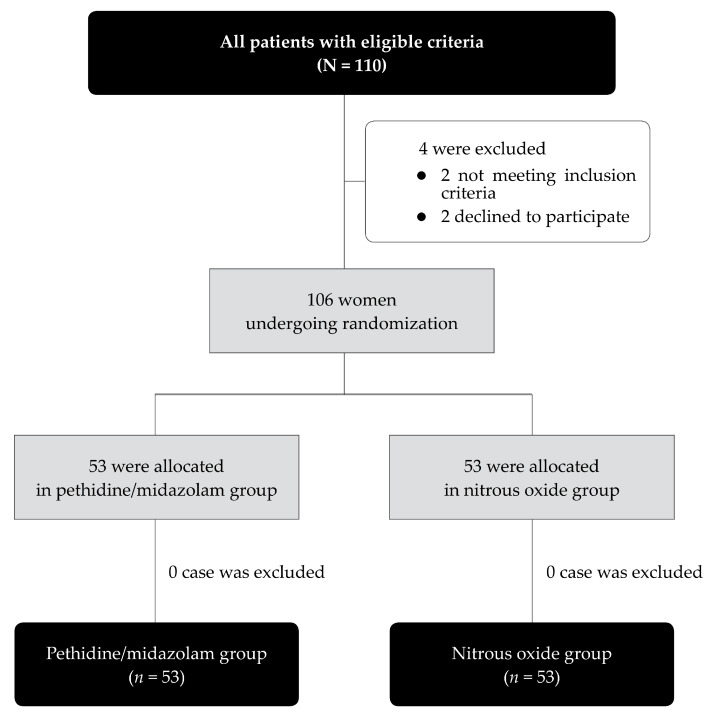
Flow chart showing enrollment and randomization of the study.

During the study period, August 2021 to December 2022, a total of 110 eligible women were enrolled in the study. Of them, four patients were excluded before allocation, as presented in Figure 1. The remaining 106 participants were randomized into two groups, 53 in the nitrous oxide group and 53 in the pethidine/midazolam group. Baseline characteristics and the types of procedures were not significantly different between both groups, as presented in Table 1. The mean (±SD) operative time was comparable, 16.5 ± 10.7 min in the nitrous oxide group and 15.4 + 8.0 min in the pethidine/midazolam group (*p*-value: 0.566).

Pain scores during, immediately and 30 min after procedures were not significantly different in the two groups (4.94 ± 3.15, 2.74 ± 2.57, 1.58 ± 2.13 vs. 5.47 ± 2.80, 2.98 ± 2.70, 1.64 ± 2.70; *p*-value: 0.174, 0.634, 0.889) for pethidine/midazolam vs. nitrous oxide group, respectively as presented in Table 2. Notably, no patient in the group of nitrous oxide needed additional pain control by pethidine/midazolam.

Satisfaction scores were comparable in both groups (*p*-value > 0.05), as presented in Table 3. Most participants did not have side effects after receiving the analgesic medications. However, the rate of side effects was significantly lower in the nitrous oxide group than that in the pethidine/midazolam group (3.8% vs. 28.3%, respectively; *p*-value 0.001), as presented in Table 4. Note that drowsiness and desaturation were frequently reported among patients receiving pethidine/midazolam, indicating a more systemic response and longer duration of action. Patients with desaturation can be corrected with an open airway and oxygen cannula. None of them needed hospitalization. (Desaturation was caused by upper airway obstruction, caused by a relaxed and dropped tongue secondary to respiratory depression due to pethidine, leading to the patient’s discomfort and need for an open airway by neck repositioning to relieve the symptom by increasing ventilation).

Additionally, the discharge scores, assessed by the modified post-anesthesia discharge scoring system, showed a significantly faster recovery time in the nitrous oxide group at 60 and 90 min after the procedure (*p*-value < 0.05), whereas the scores at 120 min were comparable in both groups, as presented in Table 5.

## 4. Discussion

New insights gained from this research are as follows: (1) effectiveness of nitrous oxide and pethidine/midazolam is comparable for pain relief in minor gynecologic procedures; (2) patients’ satisfaction is also not significantly different between the two groups; (3) side effects of nitrous oxide is significantly lower when compared to those in pethidine/midazolam group; and (4) patients in nitrous oxide group had significantly faster recovery time. However, though the discharge scores were better with statistical significance in the group of nitrous oxide, one or two points of scores might not be clinically significant. Nevertheless, this study supports that inhaled nitrous oxide could be an alternative analgesic technique for minor gynecologic operative procedures, which would improve patients’ comfort and tolerance to the procedures and health care quality without serious side effects.

To the best of our knowledge, this is the first study that evaluated the effectiveness and safety of nitrous oxide as pain relief during minor gynecologic procedures. Accordingly, this study could not be perfectly compared with other previous studies on the effectiveness of nitrous oxide. Nevertheless, our finding seems in agreement with that reported by Schneider et al. [8], who demonstrated that among 72 women (36 per study arm), nitrous oxide significantly decreased pain with in-office hysteroscopic sterilization compared to oral sedation, and they suggested that nitrous oxide can be an effective pain management option for such a procedure. Likewise, Del Valle Rubido et al. [7] showed that nitrous oxide is a safe and effective analgesic technique for polypectomy office hysteroscopy compared with the paracervical infiltration and control groups. In the literature review, however, contradictory findings have been reported. For example, Thaxton et al. [9] demonstrated that nitrous oxide did not further reduce pain for women receiving intravenous sedation in second-trimester surgical abortion [9], whereas Agostini et al. [10] also showed that nitrous oxide did not reduce intraoperative or postoperative pain in elective abortions by vacuum aspiration with paracervical analgesia and intravenous paracetamol. Additionally, Kan et al. [11] found that nitrous oxide did not reduce the pain level during suction evacuation for the first-trimester pregnancy termination under conscious sedation.

Interestingly, both methods in this study were not highly effective in pain relief for minor gynecologic operations (NRS pain scores of 5–6, interpreted as moderate pain relief). Nevertheless, most patients expressed high satisfaction with the procedures. This might be associated with rapid recovery, no serious side effects, short operation time, and probably acceptability of moderate pain relief among our patients. In fact, our results are consistent with those reported by Thongrong et al. [12], who compared the effectiveness of pain relief during uterine curettage (considered a minor gynecologic operation) between the standard method (intravenous morphine) with paracervical block. They demonstrated that NRS pain scores in 32 patients who received paracervical block were not statistically different from 32 patients who received intravenous morphine (NRS score of 7 and 6, respectively; *p*-value 0.013). They conclude that paracervical block could be used as another choice for pain relief during uterine curettage. Likewise, our findings supported that nitrous oxide may be another option for such a purpose. However, because both methods for pain relief were not excellent for analgesia in minor gynecologic procedures, as indicated by the mean pain scores of no lower than 5, though acceptable by the patients, suggesting that other techniques still need to be sought.

The strengths of this study include: (1) no selection bias at recruitment. This was due to the nature of randomized controlled trials in which known and unknown confounders were equally allocated to both groups, as confirmed by the similar baseline and clinical data; (2) as conducted in a single center, the same standard protocol of care was provided to all patients; (3) exclusion of the women who had a history of prior gynecologic procedure such as curettage because it might alter the pain threshold; and (4) all main outcomes were assessed using the well-validated metric tool; NRS as well as post-anesthesia discharge scoring system.

The weaknesses of this study are as follows: (1) Because of the apparent difference in the methods of drug administration, both participants and care providers could not be blinded to the groups of study, possibly resulting in a bias in the subjective assessment of pain scoring and other outcomes. (2) The types of procedures were heterogeneous and did not include some other important gynecologic procedures such as loop electrosurgical excision procedure or hysteroscopic polypectomy, etc. (3) The sedative effects after the procedure might still persist and affect the capability in pain scoring. (4) Though the sample size was adequate for primary outcomes, it might be relatively small for some rare side effects of both medications. Of note, the pain scores in the nitrous oxide group seemed to be slightly higher, though not significantly different. Thus, it is possible that studies with larger sample sizes or high power of test might express a significance of such a small difference if it existed. Nevertheless, such a small difference, if it existed, was unlikely to have a clinical impact.

Based on our findings, because of the comparable effectiveness to intravenous sedation for pain relief and its safety as well as rapid recovery, it is reasonable to use nitrous oxide for pain control during the uncomplicated short minor gynecological procedure. Advantages of nitrous oxide include its low cost and non-burdensome training of clinic staff. Additionally, nitrous oxide sedation has a rapid onset, quickly reversible with the administration of 100% oxygen, and delivery of the gas is noninvasive. However, because of some weaknesses mentioned above, larger randomized controlled trials are needed to confirm our results. In health systems that do not have the resources for analgesia sedation or anesthesia supervised by an anesthesiologist for such minor gynecological procedures, the use of nitrous oxide appears to be a second-rate alternative.

## 5. Conclusions

Nitrous oxide is as effective as pethidine/midazolam for pain relief in minor gynecological operative procedures but associated with significantly lower side effects and significantly faster recovery time. Nevertheless, because of some limitations, further studies with larger sample sizes are needed to confirm our findings.

## Figures and Tables

**Table 1:** Baseline characteristics according to group.

	Pethidine (n = 53)	Nitrous Oxide (n = 53)	*p*-Value
Age (years): Mean ± SD	37.1 ± 10.8	34.7 ± 9.8	0.236
Weight (kg): Mean ± SD	60.6 ± 14.5	58.1 ± 10.2	0.310
Height (cm): Mean ± SD	157.0 ± 5.8	157.6 ± 6.2	0.617
Previous abortion			0.652
No: n (%)	39 (73.6)	41 (77.4)	
Yes: n (%)	14 (26.4)	12 (22.6)	
Gynecologic procedures			0.164
MVA: n (%)	36 (67.9)	43 (81.1)	
D&C: n (%)	1 (1.9)	2 (3.8)	
F&C: n (%)	16 (30.2)	8 (15.1)	
LEEP: n (%)	0 (0.00)	0 (0.00)	
Underlying disease			0.522
No: n (%)	36 (67.9)	39 (73.6)	
Yes: n (%)	17 (32.1)	14 (26.4)	
Current medication			0.587
No: n (%)	44 (83.0)	46 (86.8)	
Yes: n (%)	9 (17.0)	7 (13.2)	
Food/drug allergy			1.000#
No: n (%)	53 (100.00)	52 (98.1)	
Yes: n (%)	0 (0.00)	1 (1.9)	
Total operative time (min)	16.5 ± 10.7	15.4 ± 8.0	0.566

**Table 2:** Comparisons of pain scores between the pethidine group and nitrous oxide group.

	Pethidine (n = 53)	Nitrous Oxide (n = 53)	*p*-Value *
Pain scores during procedures	4.94 ± 3.15	5.47 ± 2.80	0.174
Pain scores immediately after procedures	2.74 ± 2.57	2.98 ± 2.70	0.634
Pain scores 30 min after the procedures	1.58 ± 2.13	1.64 ± 2.703	0.889
	**Pethidine** **(n = 53)**	**Nitrous Oxide** **(n = 53)**	***p*** **-Value #**
Pain scores during procedures	5 (2–8)	6 (4–8)	0.204
Pain scores immediately after procedures	2 (0–50)	3 (0–5)	0.615
Pain scores 30 min after the procedures	0 (0–3)	0 (0–3)	0.846

* Student’s *t* test; # Mann–Whitney U-test.

**Table 3:** Comparisons of levels of satisfaction between the pethidine group and nitrous oxide group.

	Pethidine (n = 53)	Nitrous Oxide (n = 53)	*p*-Value
Dissatisfied	0 (0.0%)	0 (0.0%)	0.728
Minimal	1 (1.9%)	0 (0.0%)	
Moderate	11 (20.8%)	9 (17.0%)	
Very satisfied	28 (52.8%)	30 (56.6%)	
Most satisfied	13 (24.5%)	14 (26.4%)	

**Table 4:** Comparisons of side effects between the pethidine group and nitrous oxide group.

	Pethidine (n = 53)	Nitrous Oxide (n = 53)	*p*-Value
No: n (%)	38 (71.7%)	51 (96.2%)	0.001
Yes: n (%)	15 (28.3%)	2 (3.8%)	
Drowsiness: n (%)	4 (7.5%)	1 (1.9%)	
Drowsiness plus oxygen: n (%)	1 (1.9%)	0 (0.0%)	
Need of open airway *: n (%)	4 (7.5%)	1 (1.9%)	
Need of open airway plus oxygen: n (%)	3 (5.7%)	0 (0.0%)	
Oxygen: n (%)	3 (5.7%)	0 (0.0%)	

* Open airway: neck repositioning to relieve the discomfort caused by upper airway obstruction, caused by relaxed and dropped tongue secondary to respiratory depression due to pethidine.

**Table 5:** Comparisons of discharge scores between the pethidine group and nitrous oxide group.

Discharge Time:	Pethidine (n = 53)	Nitrous Oxide (n = 53)	*p*-Value *
60 min: median (q1–q3)	9(8–10)	10 (9–10)	0.002
90 min: median (q1–q3)	10 (8.5–10)	10 (10–10)	0.029
120 min: median (q1–q3)	10 (10–10)	10 (10–10)	0.174

* Significant.

## Data Availability

The datasets analyzed during the current study are available from the corresponding author upon reasonable request.
